# Identification of Proteomic Markers for Monitoring Direct Toxic Liver Injury (DTLI): Profiling Hepatoprotective Effects of Plant Polyphenols

**DOI:** 10.3390/ijms27146148

**Published:** 2026-07-09

**Authors:** Alexander G. Brzhozovskiy, Savva D. Semenov, Maria N. Yurova, Alexander L. Semenov, Anna E. Bugrova, Natalia V. Zakharova, Maria I. Indeykina, Daria A. Kharina, Oxana A. Kovaleva, Alexander Y. Zherebker, Elena I. Fedoros, Alexey S. Kononikhin, Evgeny N. Nikolaev

**Affiliations:** 1 The Center for Bio- and Medical Technologies, 121205 Moscow, Russiaokovaleva504@gmail.com (O.A.K.);; 2 Centre for Applied Translational Genomics (CATG), Mohammed Bin Rashid University of Medicine and Health Sciences (MBRU), Dubai Health, P.O. Box 50505, Dubai 50505, United Arab Emirates; 3 Moscow Center for Advanced Studies, 123592 Moscow, Russia; 4 N.N. Petrov National Medical Research Center of Oncology, 197758 Saint Petersburg, Russia; 5 Emanuel Institute of Biochemical Physics, Russian Academy of Science, 119991 Moscow, Russia

**Keywords:** polyphenols, hepatoprotection, mass spectrometry, biomarkers, mice

## Abstract

The aim of this study was to examine the hepatoprotective activity of multicomponent mixtures of natural origin in the BALB/C mouse model (*n =* 59), with acute direct toxic liver injury (DTLI) induced by the administration of streptozotocin (STZ) (100 mg/kg) in combination with a high-fat and high-fructose diet (HFFD). The hepatoprotective activity of activated hydrolytic lignin (Bp-Cx-1), methanolic fraction of Bp-Cx-1 (Bp-Cx-M) and isoflavones from kudzu Pueraria lobata roots (IFL) were evaluated on molecular level using mass spectrometry (MS)-based omics technologies. Untargeted label-free DIA quantitation resulted in 7214 protein groups identification (FDR 1%) after filtering across 40 liver tissue extracts. All treatment groups were closer to the control samples on the liver proteomic landscape compared to the untreated DTLI group, with the best results shown for the Bp-Cx-M and IFL groups. In order to identify differences between specific groups, we applied the post hoc Dunn’s test and used Hedges’ g as the effect size metric, revealing 64 proteins that tended to return to their normal level after treatment. In-depth proteomic liver tissue analysis enabled us not only to reveal the main pathways such as inflammation and oxidative stress, which are in a good agreement with DTLI and non-alcoholic liver disease pathophysiology, but also to evaluate hepatoprotective activity of multicomponent mixtures of natural origin containing polyphenols and mostly associated with protein metabolism (e.g., PSMD7, HCFC1) and deubiquitination pathways (e.g., UCHL3). It is worth noting that the Bp-Cx-1 isolated methanol fraction (Bp-Cx-M) demonstrated a pronounced increased hepatoprotective activity compared to the parent material due to the enrichment with active components such as polyphenols. Consistent with the proteomic findings of restored ubiquitin–proteasome function, assessment by comet assay revealed that treatments with Bp-Cx-M and IFL significantly reduced DNA damage by 50% compared to the untreated DTLI group. The developed MS-based multi-omics approach may be implemented for the robust and high-throughput screening method during assessment of new hepatoprotective agents of synthetic or natural origin.

## 1. Introduction

Non-alcoholic fatty liver disease (NAFLD), also defined as metabolic dysfunction-associated steatotic liver disease (MASLD), is a hepatic manifestation of a systemic metabolic disorder closely associated with obesity, insulin resistance, and dyslipidemia [1,2,3,4,5]. It is the most common cause of chronic liver disease worldwide, covering a spectrum of conditions ranging from simple steatosis and inflammation to fibrosis, cirrhosis, and hepatocellular carcinoma (HCC) [6,7]. The pathophysiology of NAFLD involves insulin resistance, hepatic de novo lipogenesis, lipotoxicity, mitochondrial dysfunction, oxidative stress, and chronic inflammation [8,9,10]. Mouse models utilizing high-fat diets (HFDs) are widely used to study these mechanisms and to test potential therapeutic agents [11,12,13,14,15,16].

To accelerate disease progression and model a more severe phenotype, combination regimens incorporating a hepatotoxic insult alongside a dietary challenge can be developed. One such approach utilizes streptozotocin (STZ), an alkylating agent with direct hepatotoxic effects [17]. At high doses, STZ promotes direct, off-target cytotoxicity on hepatocytes, causing elevated bilirubin and transaminase levels [18]. When combined with a high-fat and high-fructose diet (HFFD), this regimen induces a rapid and severe form of liver injury, characterized by pronounced steatosis, oxidative stress, and inflammation [19,20]. These conditions lead to what we term direct toxic liver injury (DTLI). This model has particular translational relevance, as it mirrors the clinical scenario of acute or subacute hepatotoxic exposure and allows for the rapid assessment of hepatoprotective agents. While the DTLI model shares several pathophysiological pathways with NAFLD (e.g., oxidative stress, mitochondrial dysfunction), it is important to distinguish it as an accelerated model of toxicant-associated steatohepatitis rather than a classical chronic metabolic disease.

Our recent study provided strong indications of the high hepatoprotective activity of multicomponent mixtures of natural origin containing polyphenols in a BALB/C mice model of toxin-induced liver injury [21]. In the present work, using mass spectrometry-based omics technologies, we extend this line of investigation to the more severe DTLI model induced by a single high-dose administration of STZ (100 mg/kg) followed by a HFFD. We evaluated the hepatoprotective effects of three substances: activated hydrolytic lignin (Bp-Cx-1), its methanolic fraction (Bp-Cx-M), and isoflavones from Pueraria lobata roots (IFL). By integrating untargeted proteomic profiling of liver tissue with functional assessments of DNA damage, we aimed to delineate the molecular signatures associated with DTLI pathogenesis and to identify protein networks and pathways targeted by these natural polyphenol-containing mixtures. The developed MS-based approach may serve as a robust, high-throughput screening method for the assessment of new hepatoprotective agents of synthetic or natural origin.

## 2. Results

### 2.1. Hepatoprotective Effect In Vivo

Hepatoprotective activity of multicomponent mixtures of natural origin were tested in vivo on a direct toxic liver injury (DTLI) model in BALB/C mice. During the experiment, severe metabolic disorders developed due to pancreatic dysfunction following the administration of streptozotocin (STZ) in combination with a high-fat, high-fructose diet. Mortality counts in each group were as follows: DTLI group—five mice; DTLI + Bp-Cx-1—three mice; DTLI + Bp-Cx-M—four mice; and DTLI + IFL—six mice (Table S1).

From the first week until the end of the experiment, significant weight loss was observed in all groups that underwent DTLI induction compared to the control group (Table S1). The peak weight loss occurred during the second week for all groups, after which a trend toward weight restoration was noted, particularly in the Bp-Cx-M and IFL groups. However, significant differences in body weight between the DTLI control group and the groups receiving hepatoprotection were not observed. In summary, Bp-Cx-1 and Bp-Cx-M were found to somewhat improve the survival of animals with induced DTLI but did not impact the dynamics of weight loss.

**Figure 1:**
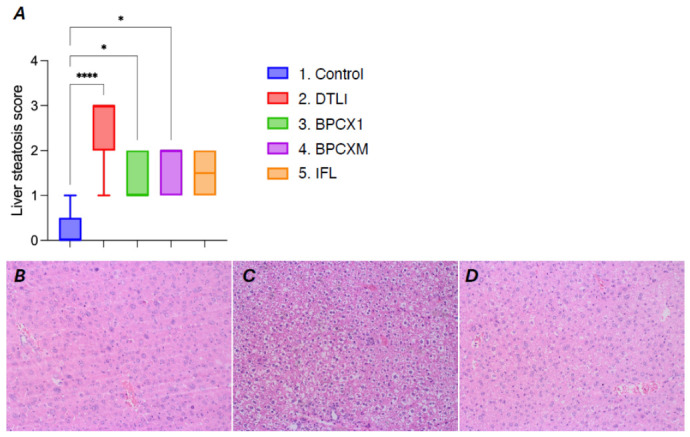
Hepatoprotective activity of naturally derived compounds in the DTLI model in BALB/C mice. (**A**) Results of scoring liver steatosis on histological sections (boxplot 95%CI, Kruskal–Wallis test with Dunn’s correction; differences are statistically significant at * *p* < 0.05 and **** *p* < 0.0001). Microphotographs of liver sections (magnification ×200, stained with hematoxylin and eosin): (**B**) control group: 0 points; (**C**) DTLI control group: 3 points; (**D**) DTLI + Bp-Cx-1 group: 2 points.

Micro/macrovesicular steatosis was observed in liver sections across all experimental groups (Table S1, Figure 1). The average steatosis index in the negative control group was 0.22 ± 0.15, while in the control DTLI group, it increased significantly to 2.43 ± 0.30 points (*p* < 0.0001). Intake of multicomponent mixtures of natural origin resulted in a reduction of the steatosis index: a 39% decrease in the IFL group (to 1.50 ± 0.22 points, *p* = 0.0681 vs. control), a 41% decrease in the Bp-Cx-1 group (to 1.44 ± 0.18 points, *p* = 0.0409), and a 43% decrease in the Bp-Cx-M group (to 1.16 ± 0.18 points, *p* = 0.0147).

A significant decrease in serum albumin and globulin levels was observed in all experimental groups compared to the negative control (Table S2). The levels of aspartate aminotransferase (AST) and alanine aminotransferase (ALT) increased significantly—by 1.5 times—in the DTLI group compared to the negative control. In contrast, the intake of multicomponent mixtures of natural origin resulted in significant reduction of these indicators toward control levels. A similar trend was noted for both total and conjugated bilirubin levels.

A tendency for the calcium to magnesium ratio to increase was observed in the DTLI group. In contrast, the intake of Bp-Cx-M led to a significant decrease in this ratio. Additionally, the creatinine level was significantly reduced in the DTLI, while the alkaline phosphatase content was significantly higher compared to the controls across all experimental groups.

### 2.2. Proteomic Analysis

**Figure 2:**
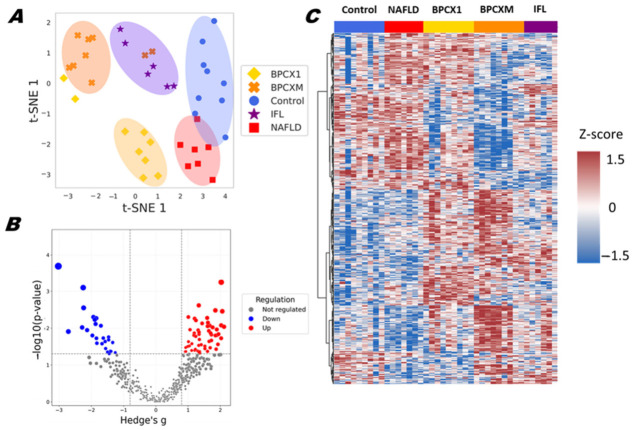
Liver proteomic landscape changes for the control, DTLI, and DTLI mouse groups with three types of treatment (BPCX1, BPCXM, IFL): (**A**) clusterization by t-SNE of liver proteomic data for all 40 samples. Colored dots depict the liver samples: control—red; DTLI—blue; BPCX1—yellow; BPCXM—orange; IFL—violet. (**B**) Volcano plot, representing proteins that were significantly upregulated or downregulated in DTLI group (*p*-value < 0.05; Hedges’ g effect size > 0.8). (**C**) Heat map of the significantly changed liver proteins (FDR < 0.05) based on the z-scores of the normalized LFQ values. The strength of the colors indicates the relative abundance of the protein in different groups.

Untargeted label-free quantitation (LFQ) was used to evaluate the changes in proteomic composition of liver tissue samples collected on the 35th day. As a result of proteomic analysis of 40 liver extracts, 8088 protein groups were identified (FDR 1%) using DIA NN software. Initially, only those features present in 80% of the samples were selected for further analysis, resulting in 7214 features (89.19%) (Table S3). To assess the changes in the proteome composition between different treatment groups, Kruskal–Wallis tests with FDR correction (FDR < 0.05) were used at a significance level of q < 0.05; as a result, 473 proteins were identified (Figure 2C). The T-SNE dimensionality reduction method shows the distribution of samples into major clusters (Bp-Cx-M, Bp-Cx-1 and other groups) (Figure 2A). The annotation of the 473 significantly changed proteins using the GO database revealed their participation in immune system pathways (68 proteins), in particular in innate immune system pathways (43 proteins) such as neutrophil degranulation (26 proteins). Several proteins also participated in apoptosis (Bcap31, Dynll2, Fas, Gsn, Oma1, Hmgb1, Ppp3r1, Gas2, Ptk2), as well as response to stress (29 proteins) and chemical stress (17 proteins). Notably, some of significantly changed proteins were involved in antioxidant activity (Sod2, Ccs, Ptgs1, Fabp1, Alox5ap, Gpx1, Gpx3, Selenot, Prdx6b, Txnrd2, Gstt2), as well as oxidative stress response (Sod2, Gpx1, Gpx3, Txn2, Txnrd2, Gstt2) and redox pathways (Sod2, Cybb, Gclm, Txn1, Ptgs1, Gpx1, Gpx3, Prdx6b, Txnrd2, Gstt2).

**Figure 3:**
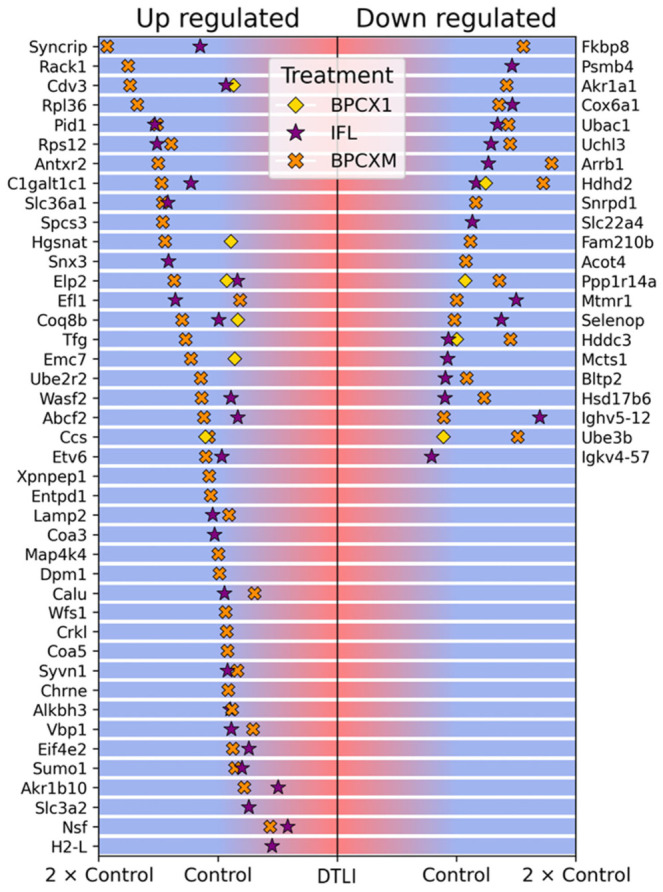
The effect of treatment assessment via analysis of the relative concentrations of differentially expressed liver tissue proteins in the DTLI mouse groups in comparison with the control group. The relative concentrations for up- and downregulated proteins were calculated as described in Methods (Section 4.3.3, Data Analysis).

In order to identify differences between specific groups, we applied the post hoc Dunn’s test (Table S4) and used Hedges’ g as the effect size metric (*p*-value < 0.05; Hedges’ g effect size > 0.8). Specifically, this approach allowed us to identify 83 proteins that differed significantly between the negative and positive control groups (Figure 2B). The levels of 64 of these proteins tended to return to normal with the treatments used (Figure 3). To demonstrate the strength of the treatment effect, we constructed a graph illustrating the change in the relative concentration of proteins under the treatment (Figure 3). Those proteins participate in protein metabolism (16 proteins), specifically in post-translational protein modification (13 proteins). In total, 238 proteins identified in the treatment groups were significantly different from the untreated DTLI. They included proteins participating in cellular response to stress (Atp6v1b2, Sod2, Dnajb6, Cybb, Surf1, Dynll2, Cox14, Dctn2, Txn1, Dctn3, Hspb8, Psmd8, Gpx1, Gpx3, Txn2, Lamtor3), in response to chemical stress (Sod2, Cybb, Surf1, Cox14, Txn1, Psmd8, Gpx1, Gpx3, Txn2), in the detoxification of reactive oxygen species (Sod2, Cybb, Txn1, Gpx1, Gpx3, Txn2), as well as in the hydrogen peroxide metabolic process (Sod2, Cybb, Hbby, Gpx1, Gpx3, Prdx6b) and cell redox homeostasis (Txn1, Ero1lb, Gpx1, Txn2, Prdx6b). For an even greater number of proteins, significant differences were found relative to both control groups in any treatment, although the control groups themselves did not differ in these proteins. Changes in levels of 106 proteins were reproducible across the different treatments used, with 65 significantly increased and 41 decreased proteins relative to both control groups. These proteins are essentially involved in cytoplasmic ribosomal process, glutathione peroxidase activity, mitochondrial respiratory chain complex IV assembly and proton-transporting ATP synthesis, glucocorticoid biosynthesis, etc.

### 2.3. Evaluation of Potential Antigenotoxic Action of Nature-Derived Polyphenols In Vivo Study

Additionally, studies of the potential antigentoxic action of multicomponent mixtures of natural origin was performed for the DTLI model. The studies were performed using an alkaline version of the DNA comet assay (for the detection of single-strand breaks and alkali-labile sites or double-strand breaks).

**Figure 4:**
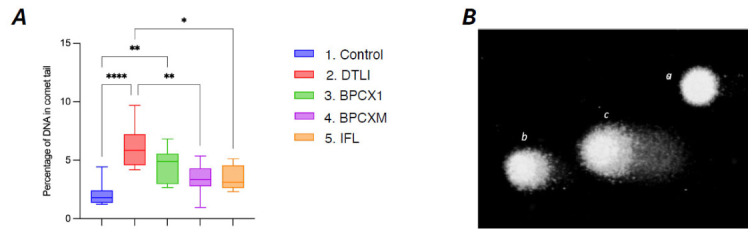
Results of DNA comet assay in liver cells in the control, DTLI, and DTLI mouse groups with three types of treatment (Bp-Cx-1, Bp-Cx-M, IFL). (**A**) Percentage of comet tail DNA in hepatocytes of female BALB/C mice upon DTLI induction. Boxplot 95%CI, Kruskal–Wallis test with Dunn’s correction; differences are statistically significant at * *p* < 0.05, ** *p* < 0.01, and **** *p* < 0.0001. (**B**) Micrographs of comets (magnification ×200, stained with SYBR Green) showing cells with intact (a) and damaged (b,c) DNA.

The results of the DNA comet assay (Figure 4) indicate a fourfold increase in DNA damage in liver cells from the DTLI control group compared to the negative control group (8.2 ± 0.77 vs. 2.1 ± 0.27, *p* < 0.0001). The administration of Bp-Cx-1 resulted in a slight reduction in the severity of STZ-induced damage (5.8 ± 0.61, *p* = 0.0806 compared to group 2). In contrast, the treatments with Bp-Cx-M and IFL significantly reduced DNA damage by 50% compared to the DTLI control group, with values of 4.2 ± 0.59 (*p* = 0.0008) and 4.4 ± 0.86 (*p* = 0.0037), respectively.

## 3. Discussion

Although the original experimental design aimed to reproduce the metabolic features of NAFLD, the administration of a high STZ dose (100 mg/kg) resulted in the establishment of a direct toxic liver injury (DTLI) model. The model demonstrated that the examined multicomponent mixtures of natural origin have a significant hepatoprotective effect. In a previous study of hepatoprotective activity of nature-derived polyphenols we suggested that isolation of the least polar fraction from Bp-Cx-1 may increase its hepatoprotective activity [22]. The proteomic analysis presented herein reveals new insights into the molecular mechanisms underlying the hepatoprotective activity of these multicomponent mixtures. The PCA analysis demonstrated a clear cluster of DTLI and samples after treatment with nature-derived polyphenols. Changes occurring during DTLI were mostly associated with metabolism of the proteins, notably Psmd7, Hcfc1. Uchl3 was among the significantly changed proteins in deubiquitination pathways that are involved in DTLI pathophysiology and have also been implicated in NAFLD [23,24]. Those proteins belong to the ubiquitin C-terminal hydrolases. Psmd7 plays a role in removing misfolded or damaged proteins [25], while Uchl3 controls levels of cellular ubiquitin through the processing of ubiquitin precursors and ubiquitinated proteins [26].

The use of Bp-Cx-1, particularly the isolated methanol fraction (Bp-Cx-M), increased the survival rate of experimental animals, reduced the degree of liver damage, and helped normalize the levels of AST, ALT, and bilirubin in the blood serum of the animals. Significantly changed proteins were involved in the oxidant detoxification process and demonstrate a pronounced antioxidant activity (Sod2, Ccs, Ptgs1, Gpx1, Gpx3, Prdx6b, Gstt2). Superoxide dismutase, mitochondrial (Sod2), is responsible for the regulation of superoxide radical anion by catalyzing the dismutation of superoxide to H_2_O_2_ [27]. Glutathione peroxidase 1 and 3 reduce small soluble hydroperoxides such as H_2_O_2_ and protects cells and enzymes from oxidative damage [28,29]. The identified changes in the regulation of proteins involved in the assembly of cytochrome c oxidase (Surf1, Oma1, Cox14) and in the proton-transporting ATP synthase complex activity (Atp5d, Vdac1, Dmac2l) also indicate a significant restructuring of the oxidative phosphorylation processes in mitochondria. Even though our model represents toxic liver injury rather than chronic metabolic disease, the proteomic alterations affecting mitochondrial homeostasis converge with mechanisms that are well documented during NAFLD [14], and therefore, their correction can achieve good therapeutic results. Overall, it should be emphasized that the majority of proteins whose regulation was significantly altered by treatment did not differ between the control and untreated DTLI groups at baseline.

This result suggests that the observed hepatoprotective effect was achieved through the stimulation of compensatory mechanisms. Such changes can be associated with the hepatoprotective activity of BP-Cx compounds. While the isoflavonoids did not impact the survival of the mice, they otherwise exhibited a similar hepatoprotective effect. Annotation of significantly changed proteins during DTLI revealed their participation in the process of GPI-anchor biosynthesis activation (MPDU1, PIGW, UBE3B, PIGQ, DPM1), which overlaps with pathways dysregulated in human NAFLD/MASLD, suggesting shared molecular nodes between toxic and metabolic liver injury [30]. Proteomic analysis also confirmed the involvement of well-established pathways in DTLI pathogenesis, such as oxidative stress and mitochondrial dysfunction. Additionally, an antigenotoxic effect was observed in the studied compositions, which was particularly pronounced in the methanol fraction of BP-Cx and the isoflavonoids. The use of these components reduced the level of DNA damage, as indicated by a twofold decrease in the amount of DNA present in the comet tail of hepatocytes from the experimental animals.

The hepatoprotective effects of multicomponent mixtures, previously identified in an in vivo model of subacute liver damage induced by carbon tetrachloride (CCl_4_), were confirmed in a model of NAFLD induced by a combination of STZ and HFFD [22]. In our research, we applied a 100 mg/kg dose of streptozotocin in accordance with early studies published by Lo at al., Almalki et al. and Zheng et al. [20,31,32]. However, this regimen resulted in significant complications, including notable animal mortality and an increase in the steatosis index from 1.93 ± 0.22 (with CCl_4_) to 2.43 ± 0.30 points. It is worth noting that the consecutive administration of lower doses of streptozotocin (30 mg/kg) has been reported to reduce lethality in experimental NAFLD models [33]. Additionally, both microvesicular and macrovesicular steatosis developed, whereas only microvesicular steatosis was previously recorded with CCl_4_. Given the severity of the hepatic insult and the direct cytotoxic effects of high-dose STZ, we have termed this experimental condition direct toxic liver injury (DTLI) to distinguish it from classic metabolic NAFLD models. In the CCl_4_ model, a statistically significant decrease in liver damage was observed with the use of Bp-Cx-1, while complete normalization of the indicator value was achieved with IFL. In the more severe DTLI model, the significant reduction of steatosis (43%) index was observed in the Bp-Cx-M group.

In the subacute model using CCl_4_, no significant changes in protein metabolism parameters were observed. However, there was an increase in the enzyme content, particularly for ALT. In the DTLI model, the levels of the main protein fractions changed, and increases were noted in both ALT and AST, as well as in total and direct bilirubin. This indicated that the studied multicomponent mixtures had a normalizing effect on these parameters. The limitations of the present study should be also acknowledged. The DTLI model induced by high-dose STZ and HFFD is acute/subacute in nature and does not recapitulate the progressive fibrosis and cirrhosis characteristic of chronic human liver disease. Overall, among the tested multicomponent mixtures, the methanol fraction of hydrolytic lignin (Bp-Cx-M) exhibited the most pronounced and multifaceted hepatoprotective profile, encompassing improvements in survival, steatosis index, biochemical markers, and DNA integrity. This enhanced activity is likely attributable to the enrichment of polyphenolic constituents achieved during fractionation. These findings support the further development of Bp-Cx-M as a promising candidate for hepatoprotective interventions. Thus, the DTLI model established on the background of HFFD may be regarded as an accelerated model of toxicant-associated steatohepatitis.

The proteomic signatures identified in this study extend beyond the characterization of hepatoprotective mechanisms in the DTLI mouse model and hold considerable translational relevance. Notably, UCHL3 is an integral component of the ubiquitin–proteasome system (UPS) and has been implicated in the development and progression of metabolic dysfunction-associated steatotic liver disease (MASLD) [34]. Furthermore, oxidative stress biomarkers SOD2, GPX1, and GPX3 play an important role in antioxidant defense impairment in human MASLD and toxic liver injury [35]. SOD2, in particular, is an important mitochondrial antioxidant that localizes in the mitochondrial matrix, where it prevents oxidation of mtDNA and protects mtDNA polymerase from being oxidized [36]. Downregulation of SOD2 expression leads to mitochondrial dysfunction, chronic inflammation, fibrosis, and increased susceptibility to liver diseases such as NAFLD [35]. Those proteins (Table 1) represent promising candidates as biomarkers for monitoring liver injury and therapeutic response in clinical settings. Validated multiple reaction monitoring (MRM) panels can be utilized for the minimally invasive quantification of these biomarkers in mouse and human plasma [37,38,39]. Notably, several of the proteins of interest have been validated by orthogonal methods in the context of liver disease. Alterations in GPX1 and SOD2 levels have been previously confirmed by Western blot and PCR analysis [21,40].

Several limitations of the present study should be acknowledged. First, the DTLI model induced by high-dose STZ and HFFD is acute/subacute in nature, meaning that it does not fully recapitulate the progressive fibrosis and cirrhosis characteristic of chronic human NAFLD/MASLD. While this model was selected for the rapid screening of hepatoprotective efficacy, further evaluation in models of chronic liver disease (CLD) is warranted. Second, although the proteomic workflow employed stringent statistical criteria (FDR < 0.05, Hedges’ g > 0.8) and was supported by orthogonal readouts (serum biochemistry, histopathology, and comet assay), the candidate protein changes were not independently validated via targeted methods such as Western blotting or qPCR. This remains a common constraint in discovery-based proteomics, defining a critical direction for future confirmatory research. Third, the experimental design lacked a standard reference drug control (e.g., silymarin). Although the primary objective was a direct comparative assessment among the three novel natural mixtures against the untreated DTLI group, incorporating a reference hepatoprotective agent will be essential for future preclinical studies. Additionally, conducting the proteomic analysis on whole liver tissue homogenates may have obscured cell-type-specific changes. Future studies utilizing spatial proteomics or single-cell approaches will provide deeper insights into the precise cellular mechanisms of action.

Despite these limitations, our integrated multi-omics approach establishes a robust framework for identifying molecular signatures of hepatoprotective activity and potential biomarkers for acute liver injury.

## 4. Materials and Methods

### 4.1. Chemicals

1. Bp-Cx-1—water-soluble lignin derivative Bp-Cx-1 [38] (Nobel Ltd., Saint Petersburg, Russia)—a sterile 0.42% ammonia solution (batch X0621D33). In the in vitro studies, Bp-Cx-1 was tested at a concentration of 0.0042% (*v*/*v*); for the in vivo studies, it was tested at a concentration of 0.42% (*v*/*v*).
2. Bp-Cx-M—methanol fraction of Bp-Cx-1 (Nobel Ltd., Saint Petersburg, Russia)—1 g of Bp-Cx-1 extracted by 100 mL of MeOH for 3 h via a Soxhlet extractor. The detailed composition of the parent BP-Cx-1 material, including its major polyphenolic constituents, has been previously characterized by MS [46].
3. IFL—NADES isoflavone extract from kudzu root (*Pueraria montana var. lobata*) (Shaanxi Sheng, Xi’an, China) [39,47]. The sample weight was 0.82 g. An alcohol extract was prepared by adding ethyl alcohol at a weight ratio of 1:2, the solution was treated with ultrasound and centrifuged at 13,000× *g* to remove the sediment. The solution was evaporated and redissolved.

### 4.2. Animal Studies

All animal studies were carried out at the center for preclinical research of the Federal State Institution “National Medical Research Center of Oncology named after N.N. Petrov” of the Ministry of Health of the Russian Federation in accordance with the Study Protocol and standard operating procedures.

The study cohort comprised 59 female BALB/C mice obtained from the Stolbovaya branch of the FSBSI “Scientific Center for Biomedical Technologies of the Federal Medical and Biological Agency” of Russia. Before the start of the experiment, animals underwent quarantine and adaptation for 17 days. Mice were housed at a room temperature of 20–23 °C, a relative air humidity of 54–58%, and an air exchange rate of 8 volumes per hour. All animals received standard complete briquetted chow (Laboratorkorm, Moscow, Russia) and filtered water ad libitum. Mice were examined daily by a veterinarian.

Approval from the Ethics Committee of the Federal State Institution “National Medical Research Center of Oncology named after N.N. Petrov” of the Ministry of Health of the Russian Federation (Protocol No 3, dated 18 March 2024) was obtained prior to all animal-related studies. All in vivo experiments were carried out in accordance with international regulations (Directive 2010/63/EU of the European Parliament and of the Council of 22 September 2010 on the protection of the animals used for scientific purposes).

#### 4.2.1. Experimental Design

Mice were randomized by weight into the negative control group (*n =* 9) and the experimental (*n =* 50) cohort.

An in vivo model of direct toxic liver injury (DTLI) in mice of the experimental cohort was induced by a single intraperitoneal injection of streptozotocin (STZ) (75% α-anomer, 98% HPLC, (CarboSynth, San Diego, CA, USA)) at a dose of 100 mg/kg followed by HFFD: pork fat added to standard chow and 30% fructose solution instead of drinking water, provided *ad libitum* throughout this study. Three days after STZ injection blood samples from the tail vein were collected for glucose level measurement using a blood glucose meter (Roche Diagnostics, Mannheim, Germany) and mice were randomized according to the results into the following groups:

1. Control—negative control—intragastric administration of placebo (drinking water) (0.2 mL/mouse) daily for 24 days (*n =* 9).
2. Control DTLI—positive control—intragastric administration of placebo (0.2 mL/mouse) daily for 24 days (*n =* 12).
3. DTLI + Bp-Cx-1—intragastric administration of Bp-Cx-1 at a dose of 80 mg/kg (0.2 mL/mouse) daily for 24 days (*n =* 13).
4. DTLI + Bp-Cx-M—intragastric administration of Bp-Cx-methanol at a dose of 80 mg/kg (0.2 mL/mouse) daily for 24 days (*n =* 13).
5. DTLI + IFL—intragastric administration of NADES isoflavone extract from kudzu root at a dose of 80 mg/kg (0.2 mL/mouse) daily for 24 days (*n =* 12).

Body weight was recorded out once a week during the experiment and prior to euthanasia.

Blood sampling was performed prior to euthanasia from the tail vein: whole blood samples were collected in 2 mL Eppendorf tubes, centrifuged (10 min, 12,000× *g*), and serum samples were immediately frozen in liquid nitrogen (−196 °C) and stored at −80 °C. Biochemical parameters of blood serum (ALT, AST, bilirubin, creatinine, urea, total protein, albumin, globulin) were evaluated with a Konelab 20 biochemical analyzer (Thermo Fisher Scientific, Waltham, MA, USA) using commercial KliniTest kits (ROO “SPbOE”, Saint Petersburg, Russia).

Euthanasia with CO_2_ was carried out on the 27th day after STZ administration. All animals were autopsied. The liver was excised and weighed, mass index (percentage ratio of liver mass to body weight) was calculated. Two liver samples from each animal were isolated: one sample was immediately used for DNA comet assay, and the other was transferred to an Eppendorf tube, frozen in liquid nitrogen, and kept at −80 °C for further analysis. The rest of the liver was examined histologically after routine histological preparation (dehydration, impregnation with paraffin, cutting into sections, staining with hematoxylin and eosin). Evaluation of liver steatosis was carried out microscopically in 10 fields (×200) per slide by a semi-quantitative method (scored as follows): 0—no changes; 1—up to 30% of hepatocytes contain lipid vacuoles in the cytoplasm; 2—30–60% of cells are changed; and 3—more than 60% of cells are changed. The mean steatosis index was calculated per animal and onward per group.

#### 4.2.2. DNA Comet Assay

Liver samples (50–200 mg) were isolated during autopsy, washed free of blood cells, ground, and a cell suspension was prepared in 1.5 mL of cooled buffer (20 mM EDTA-Na_2_, 10% DMSO, pH 7.5). A cell suspension in agarose (1:10) was immediately prepared and applied to pre-prepared gel slides with an agarose backing. Routine processing was then performed [48], including lysis, alkaline denaturation, electrophoresis, fixation, staining (with SYBR Green dye), and microscopic analysis using a microscope with a BLB-L fluorescent module (LOMO-MA LLC, Saint Petersburg, Russia). Images were digitized using an MS-20 camera (LOMO-MA LLC, Saint Petersburg, Russia) and processed using ImageJ v1.53t with OpenComet plugin v1.1 (NIH, Bethesda, MD, USA). At least 100 comets were evaluated on each slide at ×200 magnification, and the average % DNA in the comet tail was calculated.

### 4.3. Mass-Spectrometry Analysis

#### 4.3.1. Liver Samples Preparation

Liver tissue (10–20 mg) was ground into a fine powder in liquid nitrogen. Cell lysis was conducted by adding 50 mM Tris-HCl pH 8.0, 150 mM NaCl, 0.1% SDS, 0.5% Na deoxycholate, 1% NP-40 lysis buffer containing a cocktail of protease inhibitors (Roche Diagnostics, Mannheim, Germany). Tissues were incubated for 30 min and sonicated in an ultrasonic bath twice for 3 min. Samples were centrifuged at 40 °C at 10,000× *g* for 10 min, the supernatant was collected, and the sediment was re-extracted with denaturing buffer (8 M urea, 2 M thiourea, 50 mM Tris-HCl pH8.0, 0.5% NP-40). The supernatant was collected and pooled after the centrifugation at 40 °C at 10,000× *g* for 10 min. Protein concentration was measured by the Bradford method. A total of 100 μg of protein was used and precipitated with ice-cold acetone. Samples were denatured and reduced by incubation with 8 M urea, 0.1 M dithiothreitol, and 100 mM Tris × HCl (pH 8.0, +37 °C, 30 min). Next, the proteins were alkylated by 30 min incubation in the dark with 20 mM iodoacetamide. For trypsinolysis, the samples were diluted with 100 mM Tris × HCl (pH 8.0) until <1 M urea; L-(tosylamido-2-phenyl) ethyl chloromethyl ketone (TPCK)-treated trypsin (Worthington) was added at a 25:1 (protein:enzyme, *w*/*w*) ratio; and the samples were incubated for 16 h at 37 °C. The reaction was quenched by acidifying the samples with formic acid (FA) to a final concentration of 1.0% (pH ≤ 2). The resulting peptides were purified by solid-phase extraction on C18 cartridges (Waters Corp., Milford, MA, USA)), then lyophilized and dissolved for analysis in 0.1% formic acid; approximately 200 ng of peptides was injected for each LC-MS/MS acquisition.

#### 4.3.2. LC-MS/MS Analysis

Tissue tryptic peptide fractions were analyzed on a nano-HPLC (high-performance liquid chromatography) Dionex Ultimate 3000 system (Thermo Fisher Scientific, Waltham, MA, USA) coupled to a timsTOF Pro (Bruker Daltonics, Billerica, MA, USA) mass spectrometer. LC separations were performed at a flow of 400 nL/min using a 90 min linear gradient from 2% to 37% solvent B, followed by an LC column wash step (10 min isocratic with 90% solvent B) and equilibration (15 min, isocratic, with 2% solvent B) in three technical replicates. The MS data were acquired using the diaPASEF method. The electrospray source (ESI) settings were as follows: capillary voltage—1400 V; dry gas flow—3.0 L/min at a temperature of 180 °C. The MS and MS/MS spectra were acquired over an *m*/*z* range of 100 to 1700 and an ion mobility range of 0.6–1.6 1/K0 (Vs/cm^2^). The ion mobility was scanned from 0.6 to 1.6  Vs/cm^2^. The ramp time was set to 100 ms. The collision energy was ramped linearly as a function of the mobility from 59 eV at 1/K0  =  1.6 Vs/cm^2^ to 20 eV at 1/K0  =  0.6 Vs/cm^2^.

#### 4.3.3. Data Analysis

The obtained LC-MS/MS raw data were analyzed using DIA NN (Data-Independent Acquisition by Neural Networks) software (version 1.8.1) [49] in library-free mode using the following parameters: mass accuracy—20 ppm; MS1 accuracy—20 ppm; peptide length range 7–30 amino acids; search against the SwissProt Mus musculus database with carbamidomethylation (C) and oxidation (M) as possible modifications. FDR thresholds were set to 0.1%.

The statistical analysis and data visualization were performed using Python (3.7.3) with the following packages: SciPy v1.18.0 [50], Seaborn v0.13 [51], Matplotlib v3.11 [52], and Pandas v3.0.4 [53]. A Shapiro–Wilk normality test was used for each protein and each group separately. The Benjamini–Hochberg procedure was used to control the false discovery rate (FDR). As a result, we confirmed that the assumption of data normality could be accepted at the significance level q-value < 0.05. A Kruskal–Wallis test with FDR correction was used to identify potential markers between groups, followed by a post hoc Dunn’s test. To increase the test’s power and control the Type I error rate, we considered results statistically significant when *p* < 0.05 and |effect size| > 0.8. Given the small sample size and the normal distribution of the data, we used Hedges’ g as the effect size metric. Hedges’ g was chosen as the effect size metric because it corrects for bias in small sample sizes, unlike Cohen’s d. The threshold of g > 0.8 (corresponding to a ‘large’ effect size according to Cohen’s conventional criteria) was selected to identify robust, biologically meaningful changes given the relatively small group sizes (*n =* 3–6). The combination of *p* < 0.05 (after Benjamini–Hochberg correction) and Hedges’ g > 0.8 was applied to minimize false positives while retaining sensitivity to strong treatment effects. Missing values were imputed using the k-nearest neighbors method from the FancyImpute library [54], applied separately to each feature and group.

To calculate the relative concentration for Figure 3, we used the following formula: RC_{T} = (C_{T} − min(C_{C}, C_D}))/(|C_{C} − C_{D}|), where: C_{T}—mean of the protein concentration in the treatment group T; C_{C}—mean of the protein concentration in the control group; C_{D}—mean of the protein concentration in the NADFL group.

## 5. Conclusions

In summary, Bp-Cx-M demonstrated pronounced hepatoprotective activity in the DTLI model, likely due to the enrichment of active polyphenolic components. Treatments with Bp-Cx-M and IFL significantly reduced DNA damage by 50% compared to the untreated DTLI group. The MS-based multi-omics approach proved to be a powerful screening platform for assessing the hepatoprotective activity and molecular mechanisms of complex multicomponent mixtures of natural origin. While our findings are promising, further studies in models of CLD are warranted to evaluate the long-term efficacy and translational potential of these compounds. Validated multiple reaction monitoring (MRM) panels may provide a practical tool for the minimally invasive quantification of these candidate biomarkers in mouse and human plasma, supporting future translational research.

## Figures and Tables

**Table 1:** Proteomic pathways and candidate biomarkers of DTLI with relevance to NAFLD pathology.

Pathway	Potential Biomarker Proteins	Alteration in DTLI	Relevance to Human Liver Pathology
Ubiquitin–Proteasome System (Deubiquitination)	PSMD7, UCHL3, HCFC1	Dysregulation of protein degradation	Ubiquitination and deubiquitination are involved in regulation of the NAFLD pathophysiology [34,41]
Oxidative Stress Response	SOD2, GPX1, GPX3, PRDX6B, TXNRD2, GSTT2	Imbalance of pro-/antioxidant systems	Mitochondrial dysfunction and oxidative stress contribute to NAFLD progression and are key drivers of fibrosis [42,43]
Mitochondrial Homeostasis	SURF1, COX14, OMA1, ATP5D, VDAC1	Impaired respiratory chain assembly and function	Disruption of mitochondrial homeostasis occurs in the early stages of NAFLD and mitochondrial dysfunction reinforces disease progression [42,44]
Inflammation & Immune Signaling	HMGB1, FAS, PTGS1, ALOX5AP	Activation of pro-inflammatory pathways	Inflammatory activation drives the progression from simple steatosis to steatohepatitis and subsequent fibrosis [13,45]

## Data Availability

Data are contained within the Supplementary Materials.

## Supplementary Materials

The following supporting information can be downloaded at: https://www.mdpi.com/article/10.3390/ijms27146148/s1.
